# Emerging Role of Chimeric Antigen Receptor-Natural Killer Cells for the Treatment of Hematologic Malignancies

**DOI:** 10.3390/cancers17091454

**Published:** 2025-04-26

**Authors:** Ugo Testa, Germana Castelli, Elvira Pelosi

**Affiliations:** Department of Oncology, Istituto Superiore di Sanità, 00135 Rome, Italy; germana.castelli@iss.it (G.C.); elvira.pelosi@iss.it (E.P.)

**Keywords:** natural killer, chimeric antigen receptor, leukemia, lymphoma, multiple myeloma, immunotherapy, hematological malignancies

## Abstract

The clinical use of T lymphocytes engineered with chimeric antigen receptors (CARs) has revolutionized the treatment of patients with refractory or relapsed hematological malignancies. CAR natural killer (CAR-NK) cells are NK cells engineered with CARs to specifically target cell antigens expressed on the membrane of tumor cells. CAR-NK cells could offer some advantages with respect to CAR-T cells, related to their specific and innate anti-tumor activity, availability as an “off the shelf” cellular therapy, reduced costs, and improved safety. Promising efficacy of CAR-Nk cell therapy was observed in clinical trials based on the treatment of some hematological malignancies. However, to date, the clinical experience of CAR-NK cell therapy has been preliminary, with the evaluation of only a limited number of patients. Furthermore, CAR-NK cell therapy has been limited by the short persistence of these cells and by the suboptimal cytotoxic activity of some CAR-NK preparations. Therefore, studies based on the enrollment of a number of patients is required to carefully assess and confirm the safety and the efficacy of CAR-NK cell therapy in hematological malignancies and to compare their efficacy with respect to allogeneic CAR-T cells.

## Simple Summary

Natural killer (NK) cells are fundamental cellular elements of the immune system and the main effectors of the innate immune system, eliminating tumor cells without prior immunization or pre-activation. In clinical settings, allogeneic NK cell therapy has demonstrated a clear anti-leukemia efficacy and good safety, with rare induction of toxic events. Because of their intrinsic biology and favorable safety profile, NK cells have been explored as a novel cellular platform suitable for the development of adoptive cellular therapy, and recent studies have evaluated the safety and antitumor activity of NK cells engineered with chimeric antigen receptor (CAR) machinery to drive specific targeting and to enhance cytotoxicity. This review analyzes the initial clinical studies carried out using CAR-NK cells in hematological malignancies, highlighting the potential advantages of CAR-NK cells compared to CAR-T cells, but also the limitations of CAR-NK cells and the current strategies attempting to overcome these limitations.

## 1. Introduction

The capacity of the immune system to mediate the eradication of leukemic cells is strongly supported by Graft Versus Leukemia (GVL) occurring after allogeneic hematopoietic stem cell transplantation (allo-HSCT). Thus, during recent decades, numerous immunotherapy treatments have been explored in patients affected by hematological malignancies, ranging from monoclonal antibodies, either free or conjugated with cytotoxic drugs or with radioactive compounds, bispecific T-cell engagers (mediating both direct targeting of leukemic cells and driving an immune reaction of anti-leukemia), Chimeric Antigen Receptor T-cells (CAR-T), and infusions of NK cells and CAR-NK cells [1]. Some of these treatments are associated with consistent therapeutic responses in hematological patients, particularly those bearing B-cell malignancies.

The development of Chimeric Antigen Receptor T-cells (CAR-T) has provided an important contribution to the treatment of hematological malignancies, particularly for patients with refractory or relapsed disease. The basic structure of CARs involves extracellular receptors containing a single-chain variable fragment able to specifically recognize cell membrane antigens expressed on the surface of tumor cells, independent of HLA complex restriction. Each CAR molecule is constituted by four different components: an extracellular domain involved in antigen binding, a hinge region, a transmembrane region, and one or more intracellular signaling domains [2,3]. The procedure for engineering CAR-T cells has evolved over time with the development of five different CAR-T generations, with a molecular architecture progressively adapted to allow optimal proliferation, decreased cytotoxicity and increased lifespan in vivo, and a superior antitumor activity with reduced toxicity in the clinic [3]. CAR-T cells induce potent anti-tumor effects through two main mechanisms: a direct mechanism through specific targeting and consequent killing of tumor cells and an indirect mechanism mediated by the activation of a local and systemic immune anti-tumor response.

CAR-T cell therapy represents an important treatment option for a wide range of B-cell malignancies with R/R disease, inducing a high rate of responses and durable remissions in some patients. However, in spite of the very significant successes of CAR-T cell-based therapy in B-cell malignancies, associated with a less significant success in T-cell and myeloid malignancies, its widespread diffusion has been limited by some obstacles, mainly represented by its toxicity, cost, access, technological complexity, and time required for product preparation [4,5,6]. In order to bypass these limitations, CAR cell therapy based on natural killer cells (CAR-NK) is under evaluation as a valuable alternative to CAR-T cell-based therapy. As will be discussed in this review, several properties of NK cells strongly support their use for the generation of CAR-NK cells to be evaluated as an alternative to autologous CAR-T cells for the treatment of hematological malignancies.

This review will analyze recent developments in the therapy of hematological malignancies using CAR-NK cells.

## 2. Basic Biology of Human NK Cells

Natural killer (NK) cells are a type of cytotoxic cell with a key role in the context of the innate immune system. NK cells exert a cytotoxic response against virus-infected cells or tumor cells [7]. These cells are called “natural killers” because they do not require activation to kill virus-infected or tumor cells. NK cells exert their cytotoxic activity through inhibitory or activating receptors expressed on their surface [7].

The inhibitory or activating function of NK cell receptors is dictated by their intracellular domain exerting either an inhibitory or activating function on protein kinase activation. NK cell inhibitory receptors are represented by KIRs (Killer Immunoglobulin-like Receptors) and CD94/NKG2A: NKG2A recognizes non-classical MHC class I molecules such as HLA-E [1]. NK cell activation is regulated through fine control of the activity of inhibitory and activation NK cell receptors and is finalized to exert a cytotoxic activity against cells that express abnormal proteins and to tolerate self-cells.

**Figure 1:**
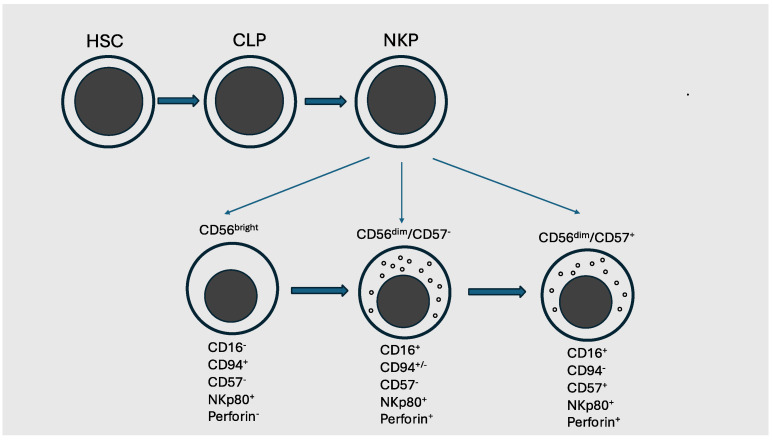
Schematic representation of human NK development. Hematopoietic stem cells generate through their differentiation common lymphoid progenitor (CLP), that in turn differentiates into NK progenitor that can seed peripheral blood and other tissues and undergo a differentiation process up to mature NK cells. Three main types of NK cells present in PB are shown: CD56^bright^, CD56^dim^/CD57^−^ and CD56^dim^/CD57^+^.

NK lymphocytes represent 5–20% of lymphocytes in blood and other sites. Two main subsets of NK are described in peripheral blood: one CD56^dim^ corresponding to 90% of total NK cells, specialized in cytolytic activity and expressing lytic granule components and a part of NK activating receptors; and the other CD56^bright^ characterized by high cytokine-secreting capacity [8]. Additional distinctions within the CD56^dim^ population are made according to the level of expression of CD57 and the absence of CD94-NKG2A expression, identifying a subset of more mature NK cells (Figure 1).

A high dimensional, single-cell study provided a more extensive characterization of these three NK cell subsets and also identified additional NK subsets, highlighting the consistent heterogeneity of these cells [9].

In addition to peripheral blood NK cells, there are also tissue-resident NK cells present in tissues, such as in the lungs, liver, lymph nodes, and uterus [8]. The maturation stage of NK cells present in the various tissues differs, and the most mature NK cells with the most pronounced effector function are observed in PB, BM, and lung tissue; precursors and immature NK cells are preferentially distributed at the level of lymph nodes and intestines and are characterized by a reduced effector function [10].

Basically, three stages of human NK cell development can be described: a first stage corresponding to lymphoid cell commitment; a second stage corresponding to commitment to NK/ILC (Innate Lymphoid Cell) lineages; and a third stage related to commitment to the NK cell lineage (Figure 1). A recent study suggested the existence of two different pathways of NK cell development in mice: one related to early NK progenitors (ENKP) differentiating into NK cells independently of common precursors for ILC (ILCPs); and the other related to ILCPs differentiating into NK cells [10]. The ENKPs mediate the generation of the equivalent of human CD56^dim^ NK cells and ILCPs determine the production of the equivalent of human CD56^bright^ NK cells [11]. These two pathways of NK cell development seem to be also maintained in humans.

The cytotoxic effects of NK cells are mediated through the formation of a synapse between NK cells and target cells, promoting activation of NK cells and secretion of the content of cytolytic granules contained in NK cells with consequent lysis of target cells, limiting bystander effects of lytic granules [8].

Several biological and functional properties of NK cells render them particularly attractive for immune anticancer therapy. In this context, particularly attractive is the capacity of NK cells to mediate lysis of tumor cells without HLA restriction and to induce a low risk of graft-versus-host disease (GVHD) when used as immunotherapy agents compared to T-cell-based immunotherapies. Thus, recent studies have evaluated two different approaches of cancer immunotherapy based on the use of human NK cells: a first approach was based on the activation and expansion of NK cells for adoptive transfer to cancer patients, while a second approach was based on a genetic engineering of NK cells inducing the expression of antibody chains driving modified NK cells (CAR-NK) specifically against tumor cells expressing a membrane antigen.

The aim of the present review consists of analyzing the results observed in recent clinical trials carried out using CAR-NK cells in patients with hematological malignancies.

## 3. NK Cell Engineering

Several recent review studies have provided a detailed analysis of the procedures used for the generation of NK-CAR cells suitable for clinical studies [12,13].

Various sources of human NK cells can be used for the generation of NK-CAR cells (Table 1). Peripheral blood NK lymphocytes represent the most used source, and these cells can be autologous or allogeneic; in the eventuality of allogeneic NK cells, T lymphocytes must be carefully removed to reduce the risk of GVHD. Alternative sources of human NK cells are represented by NK cells obtained in vitro from umbilical cord blood cells or stem cell-derived NK cells (from embryonic stem cells, hematopoietic stem cells, or induced pluripotent stem cells (iPSCs)). Each of these different sources of NK cells display some advantages and some limitations [12,13]. The different sources of human NK cells are under evaluation in current clinical trials involving CAR-NK cells.

The engineering of NK cells involves procedures similar to those previously used for the generation of CAR-T cells. Thus, similarly to CAR-T cells, the functional CAR molecule expressed in NK cells had a composition similar to that reported for CAR-T cells. Thus, essentially, the CAR molecule comprises three domains: one extracellular including a single-chain antibody fragment (scFv) mediating the recognition of the antigen; one transmembrane region connected to the extracellular domain through a hinge region and to the intracellular domain; and one intracellular domain involved in cell signaling [12,13].

## 4. CD19-CAR-NK

Several studies have supported the rationale of using CAR-NK cells targeting CD19 as a possible alternative to CD19 CAR-T cells. These studies have used NK cells obtained from different sources.

Some studies were based on NK cells obtained from cord blood. Studies carried out by Shah et al. showed that it is possible to generate human NK cells starting from CB mononuclear cell growth in the presence of a feeder of irradiated antigen-presenting cells and of IL-2; after 1 week of culture, CD3-positive cells were eliminated through immunomagnetic sorting and, after two weeks of culture, the consistent expansion of a population of functional CD56^+^/CD3^−^ human NK cells exhibiting cytotoxic activity against various tumor targets was observed [14]. These CB-derived NK cells were used in the context of a phase I study, in association with autologous HSCT in multiple myeloma patients, and their administration was well tolerated, without major safety concerns [15]. This study provided evidence that CB-derived NK cells can be safely administered to MM patients up to 1 × 10^8^ cells, thus supporting the clinical use of these cells.

More recently, an efficient cell culture system was developed for optimal expansion of human natural killer cells grown with a feeder cell support, using a cytokine cocktail (IL-2 and IL-8) and antibodies (anti-CD16 and anti-NKp46) to sustain their survival and proliferation [16]. Using these culture conditions, a consistent expansion of a population of virtually pure, functional CD56^+^/CD3^−^ NK cells was observed [16].

Wu et al. have evaluated the safety and efficacy of infusions of CB-derived NK cells after autologous HSCT and compared these to a control group not receiving NK cell infusions, observing a decrease in relapse rate (9.7% and 24.4%, respectively) and an improvement of progression-free survival (PFS) at 4 years (84.4% vs. 73.5%, respectively) and overall survival (OS) at 4 years (100% vs. 78.1%, respectively) [17]. Only mild infusion reactions were observed in 15.5% of cases.

A recent study showed that NK cells obtained in CB cultures originate from CD56^−^ progenitor cells, CD3^−^, CD14^−^, CD19^−^, HLA-DR^−^, CD34^−^, and CD7^+^ [18].

Using CB-derived NK cells, Liu et al. have reported the generation of CAR-NK cells engineered to target CD19 through transduction with a retroviral vector encoding the genes for anti-CD19 CAR, the CD28 and CD3ξ signaling endomain, interleukin-15 (IL-15), and inducible suicide gene caspase-9 [19]. In this study, CB NK cells were purified by CD3, CD19, and CD14 selection and the culture of CD3^−^, CD19^−^, and CD14^−^ cells in the presence of engineered K562 feeder cells expressing membrane-bound IL-21 and 4-1BB ligand and exogenous IL-2; on day 6, cells were transduced with the CD19 CAR vector and expanded for an additional 9 days and then harvested for fresh infusion on day 15 [19]. A total of 11 patients with CD19-positive refractory/relapsed (R/R) hematological malignancies were included in the study (5 with CLL, 6 with lymphoma, either large B-cell lymphoma (LBCL) (2 patients) or follicular lymphoma (FL) (4 patients) [19] (Table 2). The patients received a single infusion of CD19-CAR-NK cells either at 1 × 10^5^ or 1 × 10^6^ or 1 × 10^7^ cell/kg of body weight after lymphodepleting chemotherapy with fludarabine and cyclophosphamide [19]. At a median follow-up of 13.8 months, 73% of patients had an objective response, with 7/8 of responding patients achieving a complete remission (CR); 5/8 responding patients received post-remission therapy [19]. No events related to cytokine release syndrome (CRS), neurotoxicity, or GVHD were reported [19]. In vivo expansion of infused CAR-NK cells was observed as early as 3 days post-infusion and CAR-NK cells were detected for at least 12 months after infusion [19].

More recently, the final results of this trial were published, including 26 patients of the expansion cohort treated at 1 × 10^7^ CD19 CAR-NK (11 patients) or at 8 × 10^7^ CVD19 CAR-NK dose (15 patients) [20]. Thus, the final analysis of the study was based on 37 heavily pretreated patients with R/R B-lymphoid malignancies; objective responses were observed in 100% of patients with low-grade non-Hodgkin lymphoma (NHL), 67% of chronic lymphocytic leukemia (CLL) patients without transformation, and 41% of patients with diffuse large B-cell lymphoma (DLBCL); CR rates were 83% in low-grade NHL, 50% in CLL, and 29% in DLBCL [20]. Two parameters related to cord blood units used for NK cell preparation appeared to represent key determinants of the response observed: CAR-NK cells obtained from a cord blood unit (CBU) with nucleated red blood cells ≤ 8 × 10^7^ and a collection-to-cryopreservation time ≤ 24 h (“optimal CBUs) were predictors for better outcomes [20]. Only NK cells obtained from optimal CBUs were highly effective in cytotoxic activity [20]. Furthermore, trogocytosis appeared to be a predictor of relapse. Trogocytosis was defined as the process consisting of the transfer of the target antigen from the tumor to the CAR effector cells, determining a reversible transcriptional downregulation in tumor-cell antigen expression [21,22]. Furthermore, trogocytosis also causes self-recognition and fratricide by sibling cells [21,22]. The analysis of the treated patients showed that they can be subdivided into TROG^high^ exhibiting high CD19 expression on CAR-NK cells and TROG^low^ with low/absent trogocytic CD19 expression: patients with TROG^high^ had a worse 1-year OS compared to those with TROG^low^ [21,22].

A recent study reported the preliminary results on the safety and efficacy of TAK-007 in adult patients with R/R LBCL and indolent-NHL in the context of a phase II study (NCT050220015) [23] (Table 2). TAK-007 is an off-the-shelf, allogeneic, cryopreserved, CB-derived, CD19-targeting CAR-NK, produced by Tanaka Company. A total of 27 patients were enrolled into dose escalation (9 patients) and dose expansion (18 patients); two TAK-007 doses were evaluated: 200 × 10^6^ or 800 × 10^6^ cells per patient. No responses were observed in the 3 LBCL patients receiving the lower dose, while 7/14 LBCL patients receiving the higher dose had a response (21% at CR); among 9 NHL patients, 78% had a response (56% at CR) [23]. The safety profile was acceptable and mainly related to lymphodepletion chemotherapy and disease conditions; CRS was observed in three patients of grade 1 or 2 [23]. The responses seen in this trial were not particularly durable and it was suggested that the higher dose of TAK-007 (800 × 10^6^ cells) could be administered in more than one dose, thus resulting in a higher efficacy.

Another recent study reported the preliminary results on safety and efficacy of an off-the-shelf immunotherapy product obtained through the generation of anti-CD19 CAR-modified NK cells (CD19-BBz-CAR-NK) by transduction of cord blood-derived NK cells [24]. Preclinical studies have shown the efficacy of these CAR-NK cells, thus supporting a phase I clinical study (NCT05472558) aiming to assess the safety and preliminary the efficacy of repetitive administrations of CD19bBBz CAR-NK cells in R/R LBCL patients [24]. None of the nine treated patients exhibited CRS or neurotoxicity events; at day 30, the overall response rate (ORR) was 66.7%, with 55.6% of patients achieving a CR; at a median follow-up time of 12 months, the median PFS (mPFS) was 9 months and the overall survival rate was 58.3% [24].

Another study used NKX019, allogeneic human NK cells generated from healthy donor NK cells engineered with a CD19-targeting CAR-containing CD3zeta and OX40 costimulatory domain and a separate membrane bound for IL-15 activation; preclinical studies have supported a potent in vitro and in vivo cytotoxicity of NKX019 cells [25]. In phase I of this study, after three days of lymphodepletion, the patients received NKX019 at three dose levels (3 × 10^8^, 1 × 10^9^ or 1.5 × 10^9^ CAR-NK cells/dose × 3 doses on days 0, 7, 14 of a 28-day cycle [25]. A total of 19 subjects were enrolled in this study (14 NHL and 5 ALL or CLL). The treatment was well tolerated with myelotoxicity related to lymphodepletion chemotherapy and no events related to CRS neurotoxicity [25]. Significant clinical responses were observed only among NHL patients: 2/4 at 3 × 10^8^ dose level, 5/6 at 1 × 10^9^ and 3/4 at 1.5 × 10^9^ [25]. Eight patients achieved a CR, and among these patients, three with iNHL relapsed after more than 6 months of remission [25]. Enrollment along the expansion cohorts is ongoing and the 1.5 × 10^9^ cells dose in CAR-T-naïve LBCL, CAR-T-exposed LBCL, and in combination with rituximab will be evaluated.

A recent study reported the results of a phase I clinical trial investigating the safety and efficacy of CAR-NK cells generated from human NK cells derived from induced pluripotent stem cells iPSCs) [26]. This study is based on FT596, an allogeneic immunotherapy product generated from a clonal master iPSC line expressing a CD19-targeting CAR containing the NKG2D signaling domain coupled to the 2B4 and CD3ζ signaling domains, a high-affinity CD16 receptor (inducing antibody-dependent cell cytotoxicity), and an IL-15/IL-15R fusion protein promoting cytokine-independent persistence [26]. The design of the study was based on cycles of treatment, each involving lymphodepleting chemotherapy (cyclophosphamide and fludarabine), followed by FT596 administered at various doses, without (regimen A) or with Rituximab (regimen B); patients tolerating therapy and receiving some clinical benefit may receive a second cycle of treatment [26]. A total of 86 B-cell lymphoma patients were enrolled, 18 in regimen A and 68 in regimen B; these patients received a median of four lines of prior therapies and 38% received previous CAR-T cell therapy [26]. The treatment was well tolerated and the CRS of grade 1 or 2 was observed in 6% of patients in regimen A and 13% of patients in regimen B; no neurotoxicity events were reported [26]. In patients with follicular lymphoma, the ORR was 100% and the CRR 85%; after a median follow-up of 15 months, the median duration of response was 16.9 months; in patients with LBCL, the ORR was 38% and the CRR 25%, with a median duration of response not reached at a median follow-up of 9.1 months; in a subset of LBCL patients, excluding de novo DLBCL, ORR and CRR were 82% and 64%, respectively; two patients underwent hematopoietic stem cell transplantation (HSCT) in complete remission; in other B-cell lymphoma patients (CLL, mantle cell lymphoma, marginal zone lymphoma), overall response rate (ORR) was 52% and complete response rate (CRR) 26% [26]_._ Interestingly, in 20 LBCL patients who received prior CAR-T cell therapy, the CRR was 30% [26]. A total of 20 patients received a second cycle of FT596 treatment: those achieving a CR in the first cycle maintained the response in the second cycle [26].

Some experimental studies have defined new treatment strategies using CD19 CAR-T or CAR-NK cells that could improve therapeutic efficacy. Koh et al. have shown that co-treatment of CD19 CAR-T or CD19 CAR-NK cells with anti-CD19 potentiates the killing activity on tumor targets [27]. This effect results from two different mechanisms: the anti-CD19 CAR, although it interferes with tumor binding by CAR cells, favors the detachment of anti-CD19 CAR cells from target cells, thus, facilitating improved serial killing; the reduced interaction between CAR cells and tumor targets reduces CAR-induced trogocytosis [27]. The co-treatment with anti-CD19 monoclonal antibodies and CD19 CAR cells has a biphasic effect characterized by an early reduction in antitumor activity, followed by a sustained prolonged exposure to target cells [27].

Preclinical studies have shown that CD19 CAR-NK cells expressing IL-21 showed a greater proliferation in vivo and superior cytotoxicity, compared to CART-NK cells expressing IL-15 and, therefore, are candidates for clinical studies in patients with B-cell hematological malignancies [28].

A recent study reported the results of a meta-analysis of the studies performed involving CD19 CAR-NK cells, involving the cumulative analysis of four studies including a total of 81 patients with B-cell hematological malignancies (78% NHL and 22% lymphoid leukemias) [29]. The ORR was 52%, with 37% of CR [29].

## 5. NK and CAR-NK Cells in AML Therapy

### 5.1. Studies Involving NK-Expanded Cells

Several studies have explored the potential therapeutic use of expanded NK cells or CAR-NK cells in R/R AML patients.

Initial studies have explored the possible therapeutic efficacy of either autologous or allogeneic NK cells in R/R AML patients. Studies using autologous NK cells have shown only a limited therapeutic efficacy in AML patients. Initial studies showed that haploidentical, donor-related NK cells (2 × 10^7^ cells/Kg) infused to R/R AML patients after intensive lymphodepleting chemotherapy (cyclophosphamide and fludarabine) resulted in in vivo infusion of NK cells and remission in 5 out 19 patients; all patients received subcutaneous IL-2 administration after cell infusion [30]. Subsequent studies have confirmed the anti-leukemic activity of allogeneic NK cells in AML patients [31,32].

More recent studies have reported the use of allogeneic NK cells, replacing IL-2 with other cytokines such as IL-15 or IL-21 to avoid the toxic effects of IL-2 and its concurrent stimulation of host regulatory T cells. Thus, Cooley et al. showed that haploidentical NK cell infusions administered together with IL-15 induced complete remissions in 35% R/R AML patients [33]. IL-15 administration induced a better level of in vivo expansion of infused NK cells compared with previous studies using IL-2, but induced CRS when administered subcutaneously [33].

The administration of donor-derived NK cells, expanded ex vivo in the presence of membrane-bound IL-21, was safe and associated with improved relapse rate and disease-free survival [34].

A randomized clinical trial showed that the infusion after HSCT of donor-derived NK cells ex vivo expanded in the presence of IL-15 and IL-21 reduced disease progression [35]. Single-cell RNA sequencing studies showed that the patients receiving NK cell infusion showed a marked increase in memory-like cells, with a consequent expansion of CD8^+^ effector-memory T lymphocytes [35]. An updated analysis of this study showed that the PFS at 10 years was 25% and 12% for the NK group and the control group, respectively; the OS at 10 years was 29% and 12% for the NK group and the control group, respectively [36].

A phase I clinical trial evaluated the response of 12 R/R AML patients to the treatment based on six infusions of NK cells obtained from haploidentical donors and expanded in vitro using K562 feeder cells engineered to express membrane-bound IL21 and 4-1BBL; 58% for the treated patients responded to treatment with A CR; five responding patients proceeded to a haploidentical transplant from the same donor; 1-year OS for the whole group was 41.7% and 57% for patients who responded with CR [37].

### 5.2. Cytokine-Induced Memory-like Natural Killer Cells

NK cells are traditionally considered as cells mediating innate immunity. However, a consistent number of studies have shown that NK cells can acquire a memory of a prior activation event mediated by cytokine pre-activation using cytokines such as IL-15, IL-18, IL-12, and can respond more strongly upon subsequent challenges [38]. Several studies have supported the efficacy of memory-like natural killer (MLNK) cells. Human MLNK cells showed enhanced IFN-γ production and cytotoxicity against human AML blasts in vitro [39]. MLNK cells infused into mice xenotransplanted with human AML cells reduced leukemia burden and improved survival [39]. MLNK cells were used in the context of a phase I trial involving the infusion of donor NK cells obtained by activation of purified CD56^+^CD3^−^ NK cells for 12–16 h with IL-12, IL-15, and IL-18 (three patients at 0.5 × 10^6^/Kg; three patients at 1 × 10^6^/Kg; and three patients with all NK cells generated) after lymphodepletion preconditioning; after adoptive transfer, low-dose rhIL-2 was administered [30]. A total of 55% of ORR and 45% of CR + CRi rate were observed [39]. Mass cytometry analysis showed that NKG2A median expression in vivo was significantly increased after NK cell infusion in patients with treatment failure; treatment in vitro of MLNK cells with blocking NKG2A antibodies significantly increased NK cell IFN-γ production and restored a normal cytotoxic activity [40]. Berren-Elliott explored the safety and the efficacy of donor-derived MLNK cells on day +7 after HLA-haploidentical HSCT; 15 patients received for 3 weeks post-HSCT N-803 (IL-15 superagonist) [41]. The treatment was well tolerated and 87% of patients achieved a composite CR [41]. In conclusion, same-donor-derived MLNK cell infusions with 3 weeks of a IL-15 superagonist seem to improve the response to reduced-intensity conditioning haplo-HSCT for AML patients [41].

Bedmarski et al. evaluated the safety and the efficacy of donor-related MLNK cell infusions in nine pediatric AML patients relapsing after HSCT; after infusion, MLNK cells expanded in vivo and maintained their phenotype ≥ 3 months [42]. Four of eight evaluable patients achieved a CR at day 28; two patients maintained a durable response for ≥3 months with one patient in remission for ≥2 months [42].

Rutella et al. reported the development of WU-NK-101 (W-NK1), an off-the-shelf adoptive MLNK cell therapy [43]. This immunotherapy product was obtained from conventional human NK cells stimulated to express increased levels of activation markers and immune-suppressive receptors [44] and capable of bypassing immunosuppressive effects exerted by AML cells through the secretion of a variety of cytokines and chemokines able to reprogram the tumor microenvironment [45]. The phase I clinical trial (NCT05470140) explored the safety and the efficacy of WU-NK-101 cells in R/R AML patients, showing an acceptable safety profile and preliminary evidence of safety and efficacy. Nine patients received WU-NK-101 cells at dosage levels 300 × 10^6^, 900 × 10^6^, and 1.8 × 10^9^ cells, with three patients at each dose level [46]. In patients treated at higher dosages, the ORR was 50%, with only one severe treatment adverse event (grade < 3 anemia) and no severe CRS and ICANS [46]. Sequential analysis of key cytotoxic and maturation markers showed increased expression of CD16, Nkp44, 2B4, NKp80, CD45RA, and KIR receptors compared to baseline WU-NK-101 cells [46].

### 5.3. NK-CAR in AML Patients

A target for AML immunotherapy is represented by CD33, a myeloid-specific transmembrane sialic acid-binding receptor expressed at higher levels on AML blasts and leukemic stem cells but at lower levels on normal HSCs/HPCs. Preclinical studies based on blood-derived primary NK cells, engineered to express CAR-targeting CD33, have shown consistent antitumor activity in models of CD33-positive leukemias and primary AML blasts [47].

A phase I clinical study reported the results of 10 R/R AML patients undergoing treatment with CD33 CAR-NK cells obtained through engineering of cord blood-derived NK cells [47]. The patients received one or more infusions of CD33 CAR-NK cells after lymphodepleting chemotherapy; the treatment was well tolerated and 70% of patients developed grade 1 CRS [48]. By day 28 of treatment, 60% of patients achieved MRD-negative CR; the majority of responding patients relapsed within a few months [48].

Since CD33 CAR-NK cell efficacy could be limited by inhibitory receptors expressed in human NK cells (such as NKG2A) and their targets expressed on leukemic blasts (such as HLA.E), Bexte et al. generated CD33 CAR-NK cells combined with CRISPR/Cas9-mediated gene disruption of the NKG2A-encoding *KLRL1* gene; the CD33 CAR-NK-gene edited cells displayed potent anti-leukemic cell activity and transcriptional activation following AML exposure [49]. For their properties, these CAR-NK cells have the potential to bypass immune suppression in AML [49].

A recent study reported a unique approach aiming to develop CAR-NK cells enabled with the capacity to recognize multiple tumor antigens on the membrane of leukemic cells (to bypass the problem of tumor heterogeneity) and to detect the presence of a protective antigen on the membrane of healthy cells, thus avoiding their inappropriate killing due to on-target/off-tumor expression of tumor antigens [50]. This technology was explored in the context of an AML model developing CAR-NK engineered with a logic CAR to target CD33 and/or FLT3 on the membrane of leukemic cells and with a not-logic gate, with inhibitory CAR enabling CAR-NK to target endomucin, a protective antigen unique to normal HSCs [50]. This CAR-NK represented a clinical candidate, SENT-202, for the treatment of patients with hematological malignancies. On December 2024, the Senti Bio Company announced the results observed in the first three patients in the context of the results of a phase I clinical trial involving the evaluation of SENTI-202 in R/R AML patients: these first three patients were treated at the lowest dose of CAR-NK cells (1 billion cells) after lymphodepletion with Fludarabine/Cytarabine; the treatment was well tolerated with adverse events mainly related to lymphodepleting chemotherapy; 2/3 patients achieved a MRD-negative CR after one or two cycles of treatment and remained in CR at the latest follow-up (3–4 months later); 1/3 patients were resistant to the treatment [50].

Recent studies have reported the development of a new type of CAR-NK cells. This new generation of CAR-NK cells exploits the unique properties of NKT cells, a rare lymphocyte cell population present in peripheral blood and characterized by an invariant T cell receptor alpha chain and coexpression of NK markers [51]. These cells, through their TCR, recognize the non-polymorphic HLA complex-like molecule CD1d and possess potential characteristics suitable for therapeutic use since they possess tumor-killing activity and do not induce GvHD [51]. Li et al. developed a clinically guided culture method for the generation of allogeneic NKT cells and their CAR-engineered products, defined as CAR-NKT cells, through the integration of NKT TCR into human HSC and HPCs, followed by their differentiation into mature NKT cells using an ex vivo feeder-free cell culture system [51]. Li et al. recently reported the development of CAR-NKT cells engineered to target human CD33. These cells display prominently NK-like properties, with high cytotoxic activity; they kill primary AML and MDS cells with a high efficacy, through different targeting mechanisms; they target a population of leukemic cells, such as leukemia stem and progenitor cells characterized by a lower CD33 expression; and they also exhibit bone-marrow homing capacity mediated by chemokine receptors, CXCR4 and CCR5 [52]. Furthermore, these cells synergize with hypomethylating agents used in the treatment of AML and MDS [52]. Finally, these cells display a minimal off-tumor target toxicity and a low alloreactivity [52]. These properties strongly support the clinical use of CD33 NKT cells.

As mentioned above, FLT3 represents another potentially relevant target for CAR-NK cells. FLT3 is a membrane receptor frequently mutated in AML, and its targeting using CAR-T cells remains challenging for its presence on healthy HSCs/HPCs. Manson et al. reported the development of CAR-NK cells targeting FLT3, obtained through the engineering of primary human NK cells with a CAR recognizing FLT3 and secreting IL-15 [53].

The NKG2D receptor expressed on the membrane of NK cells interacts with different NKG2D ligands (NKG2DL) expressed on various types of tumor cells. NKG2DLs are expressed on bulk AML cells, but their expression is low or absent on leukemia stem cells and this was considered a potential mechanism of escaping from NK cell-mediated killing [54]. The NKX101 is an allogeneic, off-the-shelf, CAR-NK cell therapy targeting NKG2DL engineered to also express membrane-bound IL-15 [55]. Preclinical studies have shown that NKX101 exerts a marked cytotoxicity against leukemic cells expressing NKG2DL. A phase I clinical study evaluating the safety and efficacy of NKX101 in R/R AML patients showed that 4/6 patients achieved CR + CRi, with 3/6 patients having MRD negativity [55]. The treatment was well tolerated and no patient reported CRS or neurotoxicity events [55].

CD123 (IL3RA, an alpha subunit of the interleukin 3 receptor) is a well-known target for AML immunotherapy due to its higher expression on leukemic blasts compared to normal hematopoietic cells [56]. Previous studies using CAR-T cells targeting CD123 have shown target toxicities related to a side effect of normal HSCs and endothelial cells [56]. A recent study showed that these limitations of CD123-CAR-T cells can be bypassed by CD123-CAR-NK cells that do not display any significant toxicity against normal HSCs and endothelial cells [57].

Two ongoing clinical studies are evaluating the safety and the therapeutic efficacy of allogeneic CD123 CAR-NK cells in R/R AML patients (NCT05574608 and NCTo6201247). Preclinical studies have supported the evaluation of CD123 CAR-NK in combination with the trifunctional engager NKp46-CD16a-NK cell-engager targeting CD123 [58,59].

The C-type lectin-like moleculae-1 (CLL-1, CLEC12A) is a membrane antigen highly expressed on leukemic stem cells and on leukemic blasts, but not on normal HSCs. For these properties, CLL-1 is a potential target for AML immunotherapy. CLL-1 CAR-T cell therapy was evaluated in R/R AML patients reporting a high rate of ORRR 73%, with 40% of patients achieving CR + CRi); however, the treatment was associated with a consistent toxicity, with 36% of patients reporting grade 3–4 CRS and 96% of patients developing severe granulocytopenia (grade 3–4) [60]. To limit toxicity, a new CAR structure was generated by incorporating inhibitory structures CD15/CD16 to limit the effects of CAR-T on granulocytes [61].

Preclinical studies have reported that the generation of CAR-NK cells targeting CLL-1 using CAR constructs with CLL-1-specific scFv of various sizes and flexibility allowed the generation of CLL1 CAR-NK cells exhibiting cytotoxicity against primary AML blasts, but devoid of toxicity against allogeneic normal HSPCs [62]. These CLL1 CAR-NK cells are candidates for the immunotherapy of R/R AML patients [62]. Currently, three different CAR-NK trials are ongoing that involve either the use of cll1 CAR-NK alone (NCT060227853) or CLL1 together with CD33 (NCT05987696 and NCT 05215015).

### 5.4. CD70 Targeting with CAR-NK Cells

CD70 is a normally transiently expressed type 2 membrane protein on activated T-cells, B-cells, or dendritic cells, but is upregulated in many hematological malignancies and is a potential target for the treatment of these tumors.

The targeting of CD70 using monoclonal antibodies (Cusatuzumab) in association with hypomethylating agents was explored in elderly AML patients [63,64]. Preclinical studies have supported the rationale of targeting AML using CD70-specific CAR-T cells. Particularly, CD70-specific CAR-T cells were able to target AML cells, sparing normal HSCs [64]. However, CD70-specific CAR-T cells failed to completely eliminate leukemia in vivo, suggesting the need for combinatorial associations to improve the therapeutic effect [64]. In line with these observations, CD70 CAR-T cells engineered to secrete anti-CD33/anti-CD3 dual-targeting antibodies display potent anti-AML activity and are able to bypass CD70 antigen escape [65]. Recent studies have reported the development and evaluation in clinical models of CD70-targeting CAR-NK cells. Thus, Guo et al. reported the development of IL-15-secreting CD70 CAR-NK cells derived from CB mononuclear cells, exhibiting a pronounced in vitro and in vivo target-dependent cytotoxicity; repetitive administrations of CD70 CAR-NK cells resulted in durable remissions in mouse models of xenotransplanted CD19^−^ B-cell lymphomas [66]. These observations support the clinical use of CD70 CAR-NK cells, particularly in patients relapsing after CD19 CAR-T cell therapy with CD19-negative disease [66]. Another recent study, based on the development of multi-edited iPSC-derived CAR-NK cells engineered to express CD70 CAR, high-affinity non-cleavable CD16, and IL-15R/IL-15 fusion protein, showed the pronounced cytotoxic activity of these CD70 CAR-iNK cells, also exhibiting the capacity to target and eliminate recipient alloreactive T cells expressing high levels of CD70 [67].

A recent study showed that CD70 can be a feasible target for the treatment of MM patients, including those who have failed BCMA targeted therapy [68]. Thus, a preclinical study showed that MM cells, including those of patients relapsing after anti-BCMA targeted therapy, are efficiently killed by CD70 CAR-NK cells [68]. Based on the results, a phase I/II clinical trial (NCT05092451) was initiated and is recruiting patients.

### 5.5. CAR-NK Cells in Multiple Myeloma

Multiple myeloma (MM) treatment has consistently evolved and, particularly, for R/R patients, the treatment with CAR-T cells targeting the B cell receptor-maturation antigen (BCMA) was successfully introduced.

CAR-NK cells targeting various antigens expressed on the membrane of MM cells have been developed, but currently, very few patients have been treated using these agents. CAR-NK cells specifically targeting BCMA have been developed either from PB or CB and have shown an efficient killing activity against MM cells, representing potential candidates for immunotherapy in MM patients [69,70]. Alternatively, the development of BCMA-targeting CAR-NK cells using the NK-92 cell line as the NK source was reported; these cells exhibited a potent anti-MM activity both in vitro and in vivo MM models [71,72].

FT576 is a CAR-NK product based on the engineering of iPSC-derived NK cells to target BCMA. FT576 was preliminarily evaluated in the contest of a phase I study and was administered as a monotherapy or in combination with the anti-CD38 monoclonal antibody Daratumumab to R/R MM patients; the preliminary results observed in nine patients showed a good safety profile and an ORR of 22% [73].

Another study reported the development of novel NK-CAR with dual targeting capacity against BCMA and GPRC5D; dual BCM5A/GPRC5D dual-targeted CAR-NK cells improved animal survival and reduced tumor relapse compared to single-targeting CAR-NK cells [74].

Finally, a recent study reported the development of human iPSC-derived NK cells engineered to express an anti-GPRC5D CAR; the resulting off-the-shelf anti-GPRC5D iPSC-derived CAR-NK cells displayed a potent anti-MM cytotoxic activity and could represent a potential therapy for MM [75].

### 5.6. Limitations of CAR-NK Cells

Despite the consistent potentialities of NK-CAR therapies, several challenges significantly limit the expansion of their clinical applications.

In this context, a major limitation derives from their limited expansion and life span, with a short in vivo persistence. (Table 3) The activation of NK cells results in a short persistence in circulation that is no longer than 2–3 weeks [76]. To improve the survival of CAR-NK cells, various strategies have been developed, particularly those based on the use of cytokines. Essentially, these studies have shown that culturing NK cells with IL-2 and IL-15 improves expansion rates, while the presence of IL-12 and IL-21 enhances cytotoxic function [77].

To improve the survival of CAR-NK cells, various strategies have been developed, particularly those based on IL-15. IL-15 is a cytokine essential for the development and homeostasis of NK cells, memory CD8 T cells, and group 1 ILC cells. A study carried out on CB-derived NK cells transduced with a CAR19 vector showed that the incorporation of IL-15 in the transducing CAR vector resulted in enhanced in vitro and in vivo expansion and persistence in xenograft mouse models [78].

Li et al. have extended these observations through their study of NK-CAR cells engineered to express CAR19 alone, IL-15 alone, and CAR19 and IL-15: the overexpression of CAR 19 and IL-15 resulted in the generation of CAR-NK cells with increased proliferation, enhanced antitumor activity, higher metabolic fitness, improved glycolytic activity, higher MYC expression, improved persistence and survival in vivo, and longer-lasting anti-tumor response [79].

However, studies carried out in R/R AML patients receiving adoptive NK cell therapy with haploidentical cells showed that systemic IL-15 administration resulted in reduced clinical activity compared with IL-2, through a mechanism related to the promotion of recipient CD8 T-cell activation, accelerating donor NK cell rejection [80]. A possible strategy to prevent the rejection of allogeneic NK cells by the host immune cells would consist of knocking out the expression of HLA class I molecules in NK cells by gene editing [80]. On the other hand, continuous treatment with IL-15 causes exhaustion of human NK cells through the induction of a metabolic defect [81].

Another strategy to improve antitumor efficacy and in vivo persistence of CAR-NK cells consists of performing repeated, multiple infusions of these cells.

Studies carried out using CAR-T cells in hematological patients have shown that CAR-T cell levels following infusion are a very important determinant factor predicting response durability [3]. CAR-T cell levels observed during the first month of infusion are associated with improved clinical responses [3]. The extrapolation of this important observation to clinical studies with CAR-NK cells suggests that the levels of CAR-NK cells after infusion may represent one of the key factors in the anti-tumor response. However, the studies performed using CAR-NK cells involve different CAK-NK cell products with only a limited number of patients, often heterogeneous for tumor type, with a short follow-up, thus, rendering an analysis of the role of CAR-NK expansion after infusion as a determinant of the clinical response difficult. In this context, it is important to note that the only study reporting a consistent number of B-lymphoma patients treated with iPSC-derived CD19 CAR-NK cells showed a clear relationship between CAR-NK cell dose-infused and their in vivo expansion, but failed to show a clear correlation between CAR-NK expansion and anti-tumor response [26]. Studies carried out using CB-derived CAR-NK cells showed significant anti-tumor effects with CAR-NK doses of 1 × 10^6^/Kg or higher [19]. Future studies will be required to determine optimal cell doses for different types of CAR-NK cells and for different types of patients.

A potential limiting factor is represented by the difficulty in manufacturing large amounts of CAR-NK cells due to their limited in vitro expansion. Thus, among all existing sources of NK cells, CB-derived NK cells offer several advantages, including the consistent availability of UCB in cord blood banks, their non-cancerous origin, and their high proliferation potential. However, the limited number of cells available in a single CB unit and the functional immaturity of the expanded CB NK cells represent an important limitation to large-scale expansion of CB NK cells [82]. In an attempt to bypass these limitations, recent studies have reported the development of feeder-free cell culture systems for ex vivo production of NK cells from UCB suitable for immunotherapy [83,84]. Other studies have reported the development of feeder-free cell culture systems to expand and to genetically modify PB-NK cells with high proliferative capacity, preserving the responsiveness of their native activating receptors [85].

The balance between inhibitory and activating receptors determines the cytotoxic activity of NK cells; therefore, their anti-tumor activity can be potentiated either by overexpressing activating receptors or by knocking out inhibitory receptors through genome engineering, using CAR transgenesis and CRISPR-Cas9-mediated gene editing in NK cells using retroviral particles [86]. Particularly, using this technology, it was reported that the generation of CD33-specific CAR-NK cells combined with CRISPR-Cas9-based gene disruption of the NKG2A-encoding *KLRC1* gene resulted in the generation of CAR-NK cells with enhanced cytotoxicity against CD33-positive AML blasts [49]. Thus, the overcoming CAR-NK inhibition mediated by the HLA-E -NKG2A immune checkpoint induces a significant enhancement of CAR-NK-mediated cytotoxicity.

Tumor environments may exert an inhibitory activity on CAR-NK cells and may represent a significant challenge for effective immune killing. Thus, targeting cancer-associated fibroblasts represents a potentially important strategy to potentiate the antitumor activity of CARs targeting BCMA in multiple myeloma [87]. Manipulation of CAR-NK expansion conditions may reduce the inhibitory activity exerted by the tumor microenvironment [88]. Some factors released in the tumor microenvironment, such as transforming growth factor-beta (TGF-β), exert a significant immunosuppressive effect. Thus, the knockdown of TGF-β receptor II on NK cells partially overcomes the inhibitory effect of the tumor microenvironment, without affecting the anti-leukemia activity of NK cells [89].

## 6. Conclusions

CAR-NK cell therapy has the potential to address several unmet needs in the treatment of hematological malignancies. Particularly, CAR-NK cell therapy may offer several relevant advantages compared to CAR-T cell therapy, such as an improved safety, a timelier access to treatment, and reduced costs (Table 4). However, the therapeutic effects of CAR-NK cell therapy were evaluated only in a limited number of patients, with a short follow-up, and preliminary evidence suggests, in most instances, a short duration of responses. Therefore, studies in a large number of patients with a longer follow-up are absolutely required. Future studies need also to address the problem of whether single or multiple CAR-NK cell infusions are required for optimal and lasting antitumor effects. Furthermore, the various studies used different NK cell sources and it is currently unclear which NK cell source is optimal for the various subsets of hematological malignancies; the procedures of isolation and expansion of NK cells must be standardized.

Finally, CAR-NK cell therapies face some important challenges related to a short persistence of infused CAR-NK cells, limited NK cell expansion, and reduced cytotoxicity of CAR-NK cells generated from some NK cell sources. Current studies have addressed these problems and have, in part, bypassed these limitations.

Until new studies clearly support the safety and efficacy of CAR-NK cells in some hematological malignancies, this therapy will remain limited to the treatment of patients with relapsed/refractory diseases, including those who have failed autologous CAR-T cell therapy.

## Figures and Tables

**Table 1:** Characteristics of NK or NKT cells obtained from different sources. PB, peripheral blood; UCB, umbilical cord blood; hPSC, human pluripotent stem cells.

Source	Advantages	Limitations
PB-NK	Mature phenotype High cytotoxic activity	Low presence in PB Limited
UCB-NK	High proliferation Favorable safety profile	Poor differentiation Suboptimal cytotoxic activity
hPSC	Abundant availability Low immunologic risk	Safety to be evaluated Incomplete differentiation Reduced cytotoxic activity
NK-92 cell line	Abundant availability	Potential tumorigenic risk Lack expression of CD16
NKT	Mature phenotype High cytotoxic activity	Limited presence in PB Limited proliferation and expansion

**Table 2:** Main clinical studies evaluating CAR-NK cell therapy in hematological malignancies.

CAR-NK Cell Therapy	NCT Identifier	Patients (Disease and Number)	Membrane Target	NK Cell Source	Efficacy	Toxicity
CAR19/IL-15 NK cells	NCT03056339	B-cell tumors (LG-NHL, DLBCL, CLL) 37	CD19	Umbilical cord blood	ORR 49% CR 30% LG-NHL (100%; 83%) CLL (67%; 50%) DLBCL (41%; 29%)	One case CRS, no ICANS
TAK-007	NCT050220015	B-cell tumors (LBCL, NHL) 27	CD19	Umbilical cord blood	LBCL (ORR 50%; CR 21%) iNHL (ORR 78%; CR 56%)	Three cases CRS, no ICANS
CAR19bBBz NK cells	NCT05472558	B-cell tumors (R/R LBCL) 9	CD19	Umbilical cord blood	ORR 66.7% CR 55.6% mPFS 9 mo OS at 12 mon 58.3%	No CRS, no ICANS
NKX019	NCT05020678	B-cell tumors (14 NHL, 5ALL, CLL)	CD19	Peripheral blood	ORR 71% NHL ORR 77%; CR 57%	Five cases CRS, no ICANS
FT596	NCT04245722	B-cell and CLL Regimen A and B (without or with Rituximab, respectively)	CD19	iPSC	fNHL ORR 100%; CR 85% LBCL ORR 38%; CR 25%	CRS 6% reg A 13% reg B
FT576	NCT05182073	Multiple myeloma 9	BCMA	iPSC	ORR 22%	No CRS; no ICANS
NKX101	NCT04623944	AML and MDS 6	NKG2D	Peripheral blood	CR 66% CR with MRD negative 50%	No CRS; no ICANS
CD33CAR-NK cells	NCT05008575	AML	CD33	Umbilical cord blood	CR 60%	No CRS; no ICANS

**Table 3:** Main factors limiting CAR-NK cell therapy.

Limitations	Possible Solutions
Short persistence in vivo	Lymphodepleting conditioning Multiple infusions of CAR-NK cells Exogenous cytokine support Autonomous cytokine production (e.g., IL-15) Use of CIML (cytokine-induced memory-like cells) Design of activation-inhibitory CARs
Suboptimal antitumor cytotoxicity	Dual or multi-specific CAR-NK cells Gene editing of CAR-NK cells using CRISPR CAR with different affinities
Manufacturing (limited expansion)	Improvement of NK cell expansion using cytokines or feeder cells
Tumor microenvironment (TME)	Targeting of inhibitory cells in the TME Targeting of inhibitory pathways in the TME Targeting of specific NK inhibitory checkpoints

**Table 4:** Comparison of the characteristics of autologous CAR-T, allogeneic CAR-T, and allogeneic CAR-NK cell therapies.

	Autologous CAR-T	Allogeneic CAR-T	Allogeneic CAR-NK
Mechanism of antitumor effects	Specific (CAR-dependent) MHC-independent	Specific (CAR-dependent) MHC-independent	Specific and non-specific (CAR-dependent and CAR-independent) MHC independent
Antitumor efficacy	High	High	High
In vivo persistence	Long	Limited due to host-mediated immune rejection	Short
Risk of CRS or ICANS	Moderate/High	Moderate/High	Low
Risk of GvHD	No	High	Low
Off-the-shelf	No potential	High potential	High potential
Time for production	Long	Short	Short
Economic cost	High	Low	Low
Clinical efficacy	High clinical activity against hematological malignancies	High clinical activity against hematological malignancies	Promising clinical activity in some hematological malignancies
Regulatory status	Six approved products	Still under evaluation in clinical trials	Still under evaluation in early clinical use

## Data Availability

Not applicable.

## References

[1] Imai Y. (2024). Novel treatment strategies for hematological malignancies in the immunotherapy era. Int. J. Hematol..

[2] June C.H., Sadelain M. (2018). Chimeric antigen receptor therapy. N. Engl. J. Med..

[3] Cappell K.M., Kochenderfer J.N. (2023). Long-term outcomes following CAR-T cells therapy: What we know so far. Nat. Rev. Clin. Oncol..

[4] Santomasso B., Bachier C., Westin J., Rezvani C., Shpall E.J. (2019). The other side of CAR-T cell therapy: Cytokine release syndrome, neurologic toxicity, and financial burden. Am. Soc. Clin. Oncol. Educ. Book.

[5] Hoffmann M.S., Hunter B.D., Cobb P.W., Varela J.C., Munoz J. (2023). Overcoming barriers to referral for chimeric antigen receptor T cell therapy in patients with relapsed/refractory diffuse large B-cell lymphoma. Transpl. Cell. Ther..

[6] Brudno J.N., Kochenderfer J.N. (2024). Current understanding and management of CART cell-associated toxicities. Nat. Rev. Clin. Oncol..

[7] Arachchinge A.S.P.M. (2021). Human NK cells: From development to effector functions. Innate Immun..

[8] Mace E.M. (2023). Human natural killer cells: Form, function, and development. J. Allergy Clin. Immunol..

[9] Rebuffet L., Melsen J.E., Escalière B., Basurto-Lozada D., Bhandoola A., Bjorkstrom N.K., Bryceson Y.T., Castriconi R., Chichocki F., Colonna M. (2024). High-dimensional single-cell analysis of human natural killer cell heterogeneity. Nat. Immunol..

[10] Dagra P., Roncan C., Ma W., Toth M., Senda T., Carpenter D.J., Kubota M., Matsumoto R., Thapa P., Szabo P.A. (2020). Tissue determinants of human NK cell development, function, and residence. Cell.

[11] Ding Y., Lavaert M., Grassman S., Band V.I., Chi L., Das A., Harly C., Shissler S.C., Malin J., Peng D. (2024). Distinct developmental pathways generate functionally distinct populations of natural killer cells. Nat. Immunol..

[12] Zhang B., Yang M., Zhang W., Liu N., Wang D., Jing L., Xu N., Yang N., Ren T. (2024). Chimeric antigen receptor-based natural killer immunotherapy in cancer: From bench to bedside. Cell Death Dis..

[13] Yao P., Liu Y.G., Huang G., Hao L., Wang R. (2024). The development and application of chimeric antigen receptor natural killer (CAR-NK) cells for cancer therapy: Current states, challenges and emerging therapeutic advances. Exp. Hematol. Oncol..

[14] Shah N., Martin-Antonio B., Yang H., Ku S., Lee D., Cooper L., Decker W., Li S., Robinson S.N., Sekine T. (2013). Antigen presenting cell-mediated expansion of human umbilical cord blood yelds log-scale expansion of natural killer cells with anti-myeloma activity. PLoS ONE.

[15] Shah N., McCarty J., Kaur I., Yvon E., Shaim H., Muftuoglu M., Liu E., Orlowski R.Z., Cooper L., Lee D. (2017). Phase I study of cord blood-derived natural killer cells combined with autologous stem cell transplantation in multiple myeloma. Br. J. Haematol..

[16] Nakazawa T., Maeoka R., Morimoto T., Matsuda R., Nakamura M., Nishimura F., Yamada S., Nakagawa I., Park Y.S., Ito T. (2023). An efficient feeder-free and chemically-defined expansion strategy for highly purified human natural killer cells derived from human cord blood. Regen. Ther..

[17] Wu Y., Wang Y., Kuang J.J., Chen X., Liu Z., Li J., Dong T., Li X., Chen Q., Liu T. (2023). A pilot study of cord blood derived natural killer cells as maintenance therapy after autologous hematopoietic stem cell transplantation. Ann. Hematol..

[18] Wibowo T., Kogue Y., Ikeda S., Yaga M., Tachikawa M., Suga M., Kida S., Shibata K., Tsutsumi K., Murakami H. (2024). CAR-NK cells derived from cord blood originate mainly from DC56^−^CD7^+^CD34^−^HLA-DR^−^Lin^−^ NK progenitor cells. Methods Clin. Dev..

[19] Liu E., Marin D., Nanerjee P., Macalinpoc H.A., Thompson P., Basar R., Kerbany L.N., Overman B., Thall P., Kaplan M. (2020). Use of CAR-Transduced natural killer cells in CD19-positive lymphoid tumors. N. Engl. J. Med..

[20] Marin D., Li Y., Basar R., Rafei H., Daher M., Dou J., Maohanty V., Dede M., Nieto Y., Uperty N. (2024). Safety, efficacy and determinants of response of allogeneic CD19-specific CAR-NK cells in CD19+ B cell tumors: A phase 1-2 trial. Nat. Med..

[21] Hamieh M., Dobrin A., Cabriolu A.M., dan der Stegen S., Giavridis T., Manilla-Soto J., Eyquem J., Zhao Z., Whitlock B.M., Miele M.M. (2019). CAR T cell trogocytosis and cooperative killing regulate tumor antigen escape. Nature.

[22] Li Y., Basar R., Wang G., Liu E., Moyes J.S., Li L., Kerbauy L.N., Upretyy N., Fathi M., Rezvan A. (2022). KIR-based inhibitory CARs overcome CAR-NK cell trogocytosis-mediated fratricide and tumor escape. Nat. Med..

[23] Darrah J.M., Varadarajan I., Mehta A., Saultz J.N., McKinney M., Ghosh M., Gergis U., Bende G., Kasar S., Hupf B. (2024). Efficacy and safety of TAK-007, cord blood-derived CD19 CAR-NK cells, in adult patients with relapsed/refractory (R/R) B-cell non-Hodgkin lymphoma (NHL). Blood.

[24] Qian W., Lei W., Liu H., Chen W., Liang Y., Sun Y.E., Tong X., Liang A. (2024). Safety and feasibility of a 41BB co-stimulated CD19 CAR-NK cell therapy in refractory/relapsed large B-cell lymphoma. Blood.

[25] Dickinson M., Hamada N., Bryant C., Kothari N., Ojeras P., Vohra A., Lin M., Tohme M., Trager J., Shook D. (2023). First in human data of NKX019, an allogeneic CAR NK for the treatment of relapsed/refractory (R/R) B-cell malignancies. Hemasphere.

[26] Ghobadi A., Bachanova V., Patel K., Park J.H., Flinn I., Riedell P.A., Bachier C., Diefenbach C.S., Wong C., Bickers C. (2025). Induced pluripotent stem-cell-derived CD19-directed chimeric antigen receptor natural killer cells in B-cell lymphoma: A phase 1, first-in human trial. Lancet.

[27] Koh S.K., Kim H., Han B., Jo H., Doh J., Park J., Nguyen M.H., Kim H.Y., Kim H., Lee S.H. (2025). Anti-CD19 antibody co-treatment enhances serial killing activity of anti-CD19 CAR-T/-NK cells and reduces trogocytosis. Blood.

[28] He B., Chen H., Deng S., Li C., Xu N., Liu X., Zhou H., Liu Q. (2023). CD19-specific CAR NK cells coexpressing IL-21 exhibit superior expansion and antitumor activity against CD19-positive lymphoma. Blood.

[29] Carfagnini C., Singh R., Bechara S.B., Kandula M. (2024). The efficacy and safety of CD19 directed CAR-NK therapy in adults with B-cell malignancies: A meta-analysis. Blood.

[30] Miller J.S., Soignier Y., Panoskalsis-Mortari A., McNearney S.A., Yun G.H., Fautsch S.K., McKenna D., Le C., Defor T.E., Burns L.J. (2005). Successful adoptive transfer and in vivo expansion of human haploidentical NK cells in patients with cancer. Blood.

[31] Curti A., Ruggeri L., D’Addio A., Bontadini A., Dan E., Motta M.R. (2011). Successfull transfer of alloreactive haploidentical KIR ligand-mismatched natural killer cells after infusion in elderly high risk acute myeloid leukemia patients. Blood.

[32] Bachanova V., Cooley S., Defor T.E., Verneris M.R., Zhang B., McKenna D.H. (2014). Clearance of acute myeloid leukemia by haploidentical natural killer cells is improved using IL-2 diphteria toxin fusion protein. Blood.

[33] Cooley S., He F., Bachanova V., Vercellotti G.M., DeFor T.T., Curtsinger J.M., Robertson P., Grxywach B., Conlon K.C., Waldmann T.A. (2019). First-in-human trial of rhIL-15 and haploidentical naurl killer cell therapy for advanced acute myeloid leukemia. Blood Adv..

[34] Ciurea S.O., Kongtim P., Soebbing D., Trikhja P., Nehbehani G., Rondon G., Olson A., Bashir Q., Gubis A.M., Indreshpal K. (2022). Decrease of post-transplant relapse using donor-derived expanded NK cells. Leukemia.

[35] Lee K.H., Yoon S.R., Gong J.R., Choi E.J., Kim H.S., Park C.J., Yun S.C., Park S.Y., Jung S.J., Kim H. (2023). The infusion of ex vivo, interleukin-15 and -21-activated donor NK cells after haploidentical HCT in high-risk AML and MDS patients- a randomized trial. Leukemia.

[36] Lee K.H., Lee S., Park Y.H., Mun Y.C., Choi E.J., Choi Y., Park H.S., Lee J.H., Park S.Y., Yonn S.R. (2024). Interleikin-15 and -21-activated, donor-derived NK cell infusion after haploidentical HCT in high-risk AML and MDS- a cohort analysis. Leukemia.

[37] Ciurea S.O., Kongtim P., Srour S., Chen J., Soebbing D., Shpall E., Rezvani K., Nakkula R., Thakkar A., Troy E.C. (2024). Results of a phase I trial with haploidentical mbIL-21 ex vivo expanded NK cells for patients with multiply relapsed and refractory AML. Am. J. Hematol..

[38] Berrien-Elliott M.M., Wagner J.A., Fehniger T.A. (2015). Human cytokine-induced memory-like natural killer cells. J. Innate Immunol..

[39] Romee R., Rosario M., Berrien-Elliott M.M., Wagner J.A., Jewell B.A., Schappe T., Leong J.W., Abdel-Latif S., Schneider S.E., Willey S. (2016). Cytokine-induced memory-like natural killer cells exhibit enhanced responses against myeloid leukemia. Sci. Transl. Med..

[40] Berrien-Elliott M.M., Cashen A.F., Cubitt C.C., Neal C.C., Wong P., Wagner J.A., Foster M., Schappe T., Desai S., McClain E. (2020). Multidimensional analysesx of donor memory-like NK cells reveal new associations with response after adoptive immunotherapy for leukemia. Cancer Discov..

[41] Berrien-Elliott M.M., Foltz J.A., Russler-Germain D.A., Nael C.C., Tran J., Gang M., Wong P.P., Fisk B. (2022). Hematopoietic cell transplantation donor-derived memory-like NK cells functionally persist after transfer into patients with leukemia. Sci. Transl. Med..

[42] Bednarski J.J., Zimmermann C., Berrien-Elliott M.M., Foltz J.A., Becker-Hapak M., Neal C.C., Foster M., Schappe T., McClain E., Pence P.P. (2022). Donor memory-like NK cells persist and induce remissions in pediatric patients with relapsed AML after transplant. Blood.

[43] Rutella S., Vadakekolathu J., Cashen A.F., Mahajan N., Ruiz-Heredia Y., Martin-Munoz A., Barrio S., Berrien-Elliott M.M., Davidson-Moncada J., Fehniger T.A. (2023). Adoptively infused memory-like natural killer cells impact adaptive immune responses in patients with acute myeloid leukemia. Blood.

[44] Bhatnagar N., Petit V., Vadakekolathu J., Pinset C., Mahajan N., Dean J., Spingola C., Arthur L., Boocock D., Coveney C. (2023). WU-NK-101 (W-NK), a memoty-like (ML) NK cell, naturally overcomes tumor microenvironment (TME) metabolic challenges, retaining anti-tumor potency. Blood.

[45] Leedom T., Magee K., Vadakekolathu J., Arthur L., Tran M., Luukkonene L., Mahajan N., Hamil A., Muth J., Berrien-Elliott M. (2024). W-NK1 choreographs innate and adaptive immune responses to provide a robust and durable anti-AML response. Blood.

[46] Cashen A.F., Al Malki M., Stevens D.A., Muiffly L., Edwin N., Abadir E., Tan P., Niyongere S., Bajel A., Arthur L. (2024). WUN101-01: First in human (FIH) phase 1 study of WU-NK-101 (W-NK1) in patients with relapsed or refractory (R/R) acute myeloid leukemia (AML). Blood.

[47] Albinger N., Pfeifer R., Nitsche M., Merlitz S., Campe J., Stein K., Kreynberg H., Schubert R., Quadflieg M., Schneider D. (2022). Primary CD33-targeting CAR-NK cells for the treatment of acute myeloid leukemia. Blood Cancer J..

[48] Huang R., Wang X., Yan H., Tan X., Ma Y., Wang M., Han X., Liu J., Gao L., Jing G. (2025). Safety and efficacy of CD33-targeted CAR-NK cell therapy for relapsed/refractory AML: Preclinical evaluation and phase I trial. Exp. Hematol. Oncol..

[49] Bexte T., Albinger N., Al Ajami A., Wendel P., Buchinger L., Gessner A., Alzubi J., Sarchen V., Vogler M., Rasheed H.M. (2024). CRISPR/Cas9 editing of NKG2A improves the efficacy of primary CD33-directed chimeric antigen receptor natural killer cells. Nat. Commun..

[50] Frankel N.W., Deng H., Yucel G., Gainer M., Leemqns N., Lam A., Li Y., Hung M., Lee D., Banicki A. (2024). Precision off-the-shelf natural killer cell therapies for oncology with logic-gated gene circuits. Cell Rep..

[51] Li Y.R., Zhou Y., Yu J., Kim Y.J., Li M., Lee D., Zhou K., Chen Y., Zhu Y., Wang Y.C. (2024). Generation of allogeneic CAR-NKT cells from hematopoietic stem and progenitor cells using a clinically guided culture method. Nat. Biotechnol..

[52] Li Y.R., Fang Y., Niu S., Zhu Y., Chen Y., Lyu Z., Zhu E., Tian Y., Huang J., Rezek V. (2025). Allogeneic CD33-directed CAR-NKT cells for the treatment of bone marrow-resident myeloid malignancies. Nat. Commun..

[53] Mansour A.G., Teng K.Y., Li Z., Zhu Z., Chen H., Tian L., Ali A., Zhang J., Lu T., Ma S. (2023). Off-the-shelf CAR-engineered natural killer cells targeting FLT3 enhance killing of acute myeloid leukemia. Blood Adv..

[54] Paczulla A.M., Rothfelder K., Raffel S., Konantz M., Steinbacher J., Wang H., Tandler C., Mbarga M., Schaefer T., Falcone M. (2019). Absence of NKG2D ligands defines leukemia stem cells and mediates their immune evasion. Nature.

[55] Sauter C.S., Borthakur G., Muntjoy L., Rotta M., Liu H., Murthy H.S., Lin M., Trager J., Chang C., Kothari N. (2023). A phase 1 study of NKX101, a chimeric antigen receptor natural killer (CAR-NK) cell therapy, with fludarabine and cytarabine in patients with acute myeloid leukemic. Blood.

[56] Pelosi E., Castelli G., Testa U. (2023). CD123 a therapeutic target for acute myeloid leukemia and blastic plasmocytoid dendritic neoplasm. Int. J. Med. Sci..

[57] Caruso S., De Angelis B., Del Bufalo F., Ciccone R., Donsante S., Volpe G., Manni S., Guercio M., Pezzella M., Iaffaldano L. (2022). Safe and effective off-the-shelf immunotherapy based on CAR.CD123-NK cells for the treatment of acute myeloid leukemia. J. Hematol. Oncol..

[58] Gauthier L., Virone-Oddos A., Beninga J., Rossi B., Nicolazzi C., Amara C., Blanchard-Alvarez A., Gourdin N., Courta J., Basset A. (2023). Control of acute myeloid leukemia by a trifunctional NKp46-CD16a-NK cell engager targeting CD123. Nat. Biotechnol..

[59] Mendfelowitz A.S., Chu Y.Y., Felices M., Miller J., Forman S.J., Bhjbehani G., Bonifant C., Lee D.A., Cairo M. (2024). Anti-CD123 CAR NK cells and cam161533 trispecific killer have synergistic activity against acute myeloid leukemia. Blood Cancer Discov..

[60] Zhang X., Lv H., Xiao X., Bai X., Liu P., Pu Y., Meng J., Zhu H., Wang Z., Zhang H. (2023). A phase I clinical trial of CLL-1 CAR-T cells for the treatment of relapsed/refractory acute myeloid leukemia in adults. Blood.

[61] Zhang R., Zhao Y., Chai X., Wang Y., Zhao M., Guo S., Zahng Y., Zhso M. (2025). Modified CD16/CD16-CLL1 inhibitory CAR-T cells for mitigating granulocytopenia toxicities in the treatment of acute myeloid leukemia. Transl. Oncol..

[62] Sedloev D.N., Chen Q., Liglaub J.M., Schmidt A., Muller-Tidow C., Schmidt M., Sauer T. (2024). Structurally optimized CLL-1 CAR-NK cells are highly potent effectors against AML without Hpsc toxicity. Blood.

[63] Sauer T., Parikh K., Omer B., Omer B., Sedloev D., Chen Q., Angenendt L., Schliemann C., Schmitt M., Muller-Tidow C. (2021). CD70-specific CAR T cells have potent activity against acute myeloid leukemia without HSC toxicity. Blood.

[64] Wu G., Guo S., Luo Q., Wang X., Deng W., Ouyang G., Pu J.J., Lei W., Qian W. (2023). Preclinical evaluation of CD70-specific CAR T cells targeting acute myeloid leukemia cells. Front. Immunol..

[65] Silva H.J., Martin G., Birocchi F., Wehrli M., Kann M.C., Supper V., Parker A., Graham C., Bratt A., Bouffard A. (2025). CD70 CAR T cells secreting an anti-CD33/anti-CD3 dual-targeting antibody overcome antigen heterogeneity. Blood.

[66] Guo S., Lei W., Jin X., Liu H., Wang J.Q., Deng W., Qian W. (2024). CD70-specific CAR NK cells expressing IL-15 for the treatment of CD19-negative malignancy. Blood Adv..

[67] Wang L., Wang Y., He X., Mo Z., Zhao M., Liang X., Hu K., Wang K., Yue Y., Mo G. (2025). CD70-targeted iPSC-derived CAR-NK cells display potent function against tumors and alloreactive T cells. Cell Rep. Med..

[68] Lin P., Reyes Silva F.C., Lin P., Gilbert A.L., Acharya S., Nunez Cortes A.K., Banerjee P., Fang D., Melo Garcia L., Daher M. (2023). CD70 CAR NK cells in the treatment of multiple myeloma. Blood.

[69] Ren Q., Zu Y., Su H., Lu Q., Xiang B., Luo Y., Zhang J., Song Y. (2023). Single VHH-directed BCMA CAR-NK cells for multiple myeloma. Exp. Hematol. Oncol..

[70] Talarico L., Wong C., Pang C., Hickman T., Hu C., Shaw A., Wasniewski E., La S.C., Sharma P., Moore S. (2024). A cryopreserved allogeneic anti-BCMA CAR-NK cellular therapy exhibits both innate and CAR-mediated MM cell killing in vitro and in vivo. Cancer Res..

[71] Motais B., Charvatova S., Herdinka M., Hajek R., Bago J.R. (2022). Anti-BCMA-CAR NK cells expressing soluble TRAIL: Promising therapeutic approach for multiple myeloma in combination with bortezomib and γ-secretase inhibitors. Blood.

[72] Park E., Mun H.J., Seo E., Hwang S., Lee J.H., Song S., Sung H., Kim H.Y., Kwon M.J. (2024). CARNK92 tergeting BCMA can effectively kill multiple myeloma cells both in vitro and in vivo. Biomedicines.

[73] Dhakal B., Bardeja J.G., Gregory T., Ly T., Bickers C., Zong X., Wong L., Goodridge J.P., Cooley S., Valamehr B. (2022). Interim phase I clinical data of FT576 as monotherapy and in combination with daratumumab in subjects with relapsed/refractory multiple myeloma. Blood.

[74] Cao Z., Yang C., Wang Y., Wang C., Wang Q., Ye G., Liu T., Wang Q., Wang H., Gong Y. (2022). Allogeneic CAR-NK cell therapy targeting both BCMA and GPRC5D for the treatment of multiple myeloma. Blood.

[75] Yang J., Jiang L., Zhu Z., Yan Y., Fu J., Wei M. (2024). CIB315: An allogeneic, off-the-shelf anti-GPRC5D iPSC-derived CAR-NK product targeting multiple myeloma. Blood.

[76] Wu X., Matosevic S. (2022). Gene-edited and CAR-NK cells: Opportunities and challenges with engineering of NK cells for immunotherapy. Mol. Ther. Oncolytics.

[77] Maia A., Tarannum M., Lérias J.R., Piccinelli S., Borrego L.M., Mauerer M., Romee R., Castillo-Martin M. (2024). Building a better defense: Expanding and improving natural killer cells for adoptive cell therapy. Cells.

[78] Liu E., Tong Y., Dotti G., Shaim H., Savoldo B., Mukherjee M., Orange J., Wan X., Lu X., Reynolds A. (2018). Cord blood NK cells engineered to express IL-15 and a CD19-targeted CAR show long-term persistence and potent antitumor activity. Leukemia.

[79] Li L., Mohanty V., Dou J., Huang Y., Banerjee P.P., Mia Q., Lohr J.G., Vijekkumar T., Frede J., Knoechel B. (2023). Loss of metabolic fitness drives tumor resistance after CAR-NK cell therapy and can be overcome by cytokine engineering. Sci. Adv..

[80] Berrien-Elliott M.M., Becker-Hapak M., Cashen A.F., Jacobs M., Wong P., Foster M., McClain E., Deasai S., Pence P., Cooley S. (2022). Systemic IL-15 promotes allogeneic cell rejection in patients treated with natural killer cell adoptive therapy. Blood.

[81] Felices M., Lenvik A.J., McElmurry R., Chu S., Hinerlie P., Bendzick L., Geller M.A., Tolar J., Blazar B.R., Miller J.S. (2018). Continuous treatment with IL-15 exhausts human NK cells via a metabolic defect. JCI Insight.

[82] Luevano M., Daryuzeh M., Alnabhan R., Querol S., Khakoo S., Madrigal A., Saudemont A. (2012). The unique profile of cord blood natural killer cells balances incomplete maturation and effective killing function upon activation. Hum. Immunol..

[83] Corredera M.M., Paillet J., Gaudeaux P., Blein T., Sadek H., Rault P., Berriche A., Roche-Naude J., Lagreste-Peyrou C., Soheili T.S. (2025). Feder-cell-free system for ex vivo production of natural killer cells from cord blood hematopoietic stem and progenitor cells. Front. Immunol..

[84] Kunkanjanawan H., Somrendgan S., Kunkanjanawan T., Wongtrakoongate P., Wongsakmanee W., Khemarangsan V., Masuayma I., Parnpai R. (2025). Efficient large-scale expansion of cord blood-derived NK cells: Leveraging lipopolysaccharide for enhanced NK cell production. Cytotherapyy.

[85] Quintarelli C., Sivori S., Caruso S., Carlomagno S., Falco M., Boffa I., Orlando D., Guercio M., Abbaszadeh Z., Sinibaldi M. (2020). Efficacy of third-party chimeric antigen receptor modified peripheral blood natural killer cells for adoptive cell therapy of B-cell precursor acute lymphoblastic leukemia. Leukemia.

[86] Jo D.H., Kaczmarek S., Shin O., Wang L., Cowan J., McComb S., Lee S.H. (2023). simultaneous engineering of natural killer cells for CAR transgenesis and CRSPR-Cas9 knockout using retroviral particles. Methods Clin. Dev..

[87] Sakemura R., Hefazi M., Siegler E.R., Cox M.J., Larson D.P., Hansen M.J., Manriquez Roman C., Schick K.J., Can I., Tapper E.E. (2022). Targeting cancer-associated fibroblasts in the bone marrow prevents resistance to CART-cell therapy in multiple myeloma. Blood.

[88] Kilgour M.K., Bastin D.J., Lee S.H., Ardolino M., McComb S., Visram A. (2023). Advancements in CAR-NK therapy: Lessons to be learned from CAR-T therapy. Front. Immunol..

[89] Rouce R.H., Shaim H., Sekine T., Weber G., Ballard B., Ku S., Barese C., Murali V., Wu M.F., Liu H. (2016). The TGF-beta/SNAD pathway is an important mechanism for NK cell immune evasion in childhood B-acute lymphoblastic leukemia. Leukemia.

